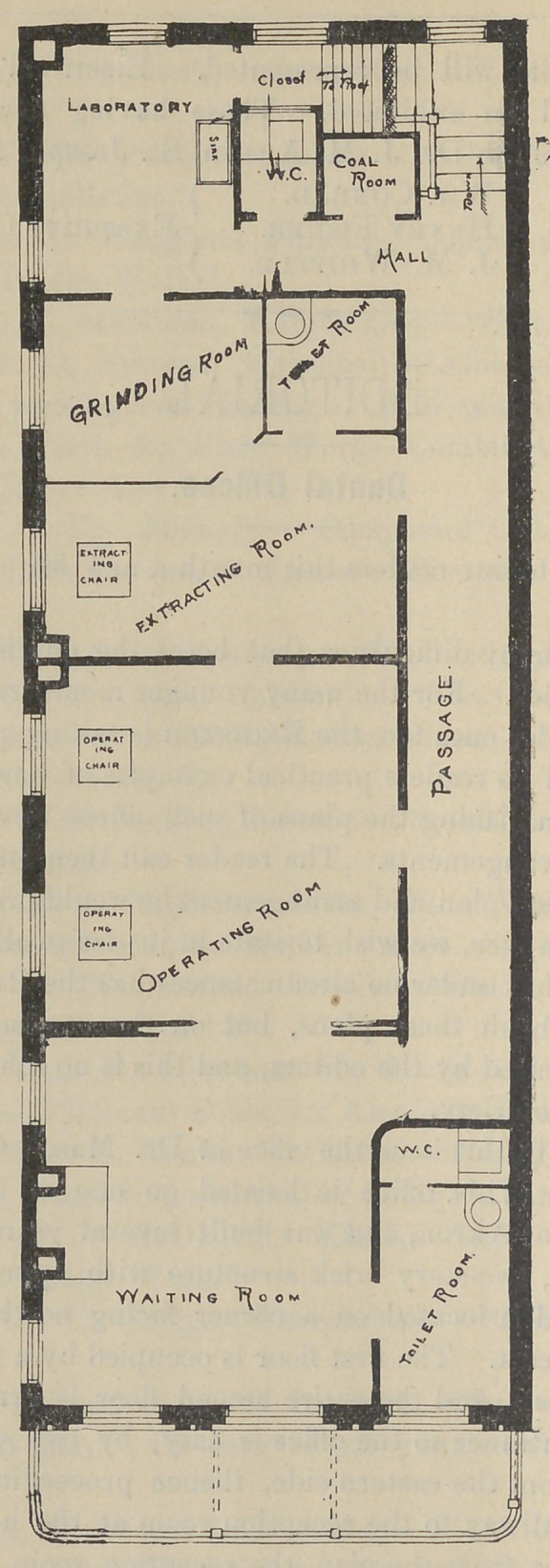# Dental Offices

**Published:** 1891-06

**Authors:** 


### Editorial.

### Dental Offices.

We present to our readers this month a new feature in dental journalism.

One of the many difficulties that beset the dentist is, how to arrange the office. For the many younger members of the profession, and older ones too, the Register is taking steps to bring to the notice of its readers practical examples of how to arrange an office, by publishing the plans of such offices as will bring in a variety of arrangements. The reader can then form his judgement as to which plan and arrangement he would prefer. In the beginning, however, we wish to state in justice to all whose plans may appear, that under no circumstances has the Register been solicited to publish these plans, but on the contrary, all plans have been solicited by the editors, and this is no scheme to bring any one into notoriety.

We present in this issue the office of Dr. Mason Chapman, of Akron, Ohio. This office is located on one of the principle thoroughfares of Akron, and was built several years ago. It is a well finished, two-story brick structure with appropriate stone trimmings, and is located on a corner facing north, whilst the side faces the east. The first floor is occupied by a merchant for business purposes, and the entire second floor is arranged as an office. The entrance to the office is easy, by two sets of stairs, approached from the eastern side, thence proceeding through a well lighted hallway to the reception room at the northern end. As may be seen from the plan, the reception room is large and

See description on next page.

commodious; it is finished with natural wood and an elegant fireplace lends attraction to the remaining part of the well decorated walls. Dr. Chapman has wisely arranged here a toilet room and closet, essentials of every dental office. The large veranda is another attractive feature in warm weather for waiting patients. The operating room is separated from the reception room by large folding doors and at this door heavy curtains are draped. The operating room is large and commodious and contains two operating chairs. Separated from this by a solid door is the extracting room with all necessary appliances, including a lounge for sick patients and near by a toilet room for the use of patients. Separated by a high partition is the grinding room with lathe for grinding teeth and a large window giving good light to work by. All the windows are of extra width and height. The laboratory includes a water-closet and coal room and a good sink. As will be seen by the plan all the rooms have entrance to the hallway which leads to the stairway thence the street. This hall is well lighted by a large stained glass window inserted at the southern end of the building opposite the hall.

This constitutes a brief description of Dr. Chapmans' office, and it must go without saying, that such an office is tastefully and well furnished with carpets and furniture. To Dr. Chapman's credit too, it may be said for the benefit of younger men, that by strict attention to business and honesty he has accumulated enough from his dental practice to furnish him with an office that any dentist might well be proud of. Dr. Chapman is widely known throughout northern and eastern Ohio and is a consistent and regular practitioner of dentistry. W.

## Figures and Tables

**Figure f1:**